# Inhibition of STAT5: A therapeutic option in BCR-ABL1-driven leukemia

**DOI:** 10.18632/oncotarget.2465

**Published:** 2014-10-09

**Authors:** Angelika Berger, Veronika Sexl, Peter Valent, Richard Moriggl

**Affiliations:** ^1^ Institute of Pharmacology and Toxicology, University of Veterinary Medicine, Vienna, Austria; ^2^ Department of Medicine I, Division of Hematology and Ludwig-Boltzmann Cluster Oncology, Medical University of Vienna, Austria; ^3^ Ludwig-Boltzmann Institute for Cancer Research, University of Veterinary Medicine, Medical University Vienna, Austria

**Keywords:** STAT5, BCR-ABL1, tyrosine kinase, leukemia, inhibitors, SH2 domain, GTPases, nuclear translocation

## Abstract

The two transcription factors STAT5A and STAT5B are central signaling molecules in leukemias driven by Abelson fusion tyrosine kinases and they fulfill all criteria of drug targets. STAT5A and STAT5B display unique nuclear shuttling mechanisms and they have a key role in resistance of leukemic cells against treatment with tyrosine kinase inhibitors (TKI). Moreover, STAT5A and STAT5B promote survival of leukemic stem cells. We here discuss the possibility of targeting up-stream kinases with TKI, direct STAT5 inhibition via SH2 domain obstruction and blocking nuclear translocation of STAT5. All discussed options will result in a stop of STAT5 transport to the nucleus to block STAT5-mediated transcriptional activity. In summary, recently described shuttling functions of STAT5 are discussed as potentially druggable pathways in leukemias.

The Janus kinase/signal transducer and activator of transcription (JAK/STAT) signaling pathway is highly conserved throughout evolution. Initial signaling starts upon ligand binding at the cytokine and growth factor receptor, emanating a signal from the cell membrane to the nucleus (Figure 1). The activation of receptor-associated tyrosine kinases – the JAKs – is followed by activation and tyrosine phosphorylation of the STATs [1]. STAT inactivation is largely carried out by tyrosine phosphatase action. Moreover, control by suppressor of cytokine signaling (SOCS), protein inhibitors of activated STAT (PIAS), signal transducing adaptor molecule (STAM), Sprouty-related Ena/VASP homology 1-domain-containing protein (SPRED) and SPROUTY proteins were described as negative regulators of JAK/STAT activation [2, 3]. STAT proteins can regulate proliferation, differentiation, growth and apoptosis [4]. In mammals, seven different STATs are known which share several conserved functional domains, but the transactivation domain (TAD) at the C-terminus is the most diverse part (Figure 2B).

**Figure 1:**
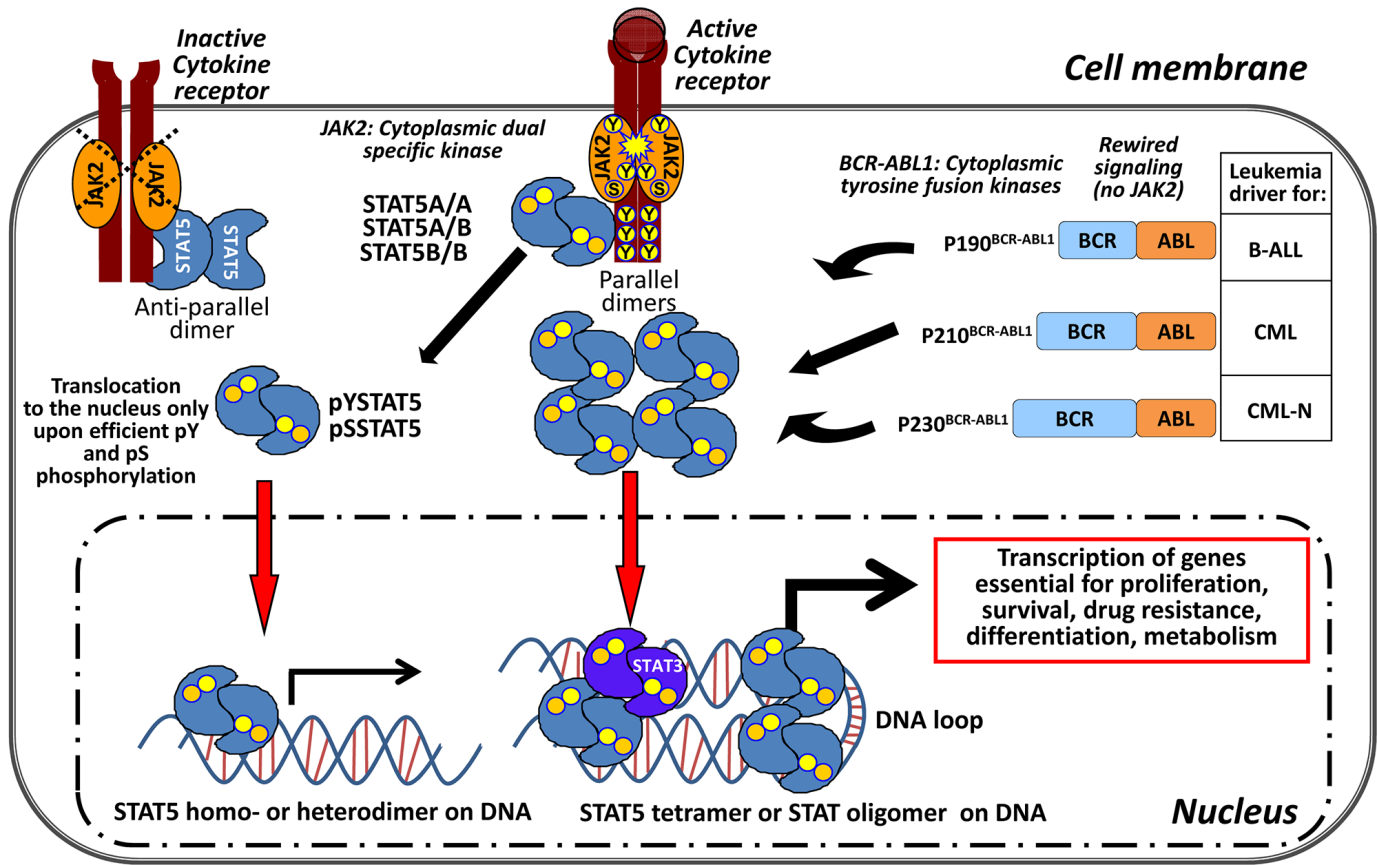
STAT5 – the central signaling node in *BCR-ABL1*^+^ leukemia Canonical activation of STAT5 starts at the cell membrane with cytokine binding to specific receptors. Receptor dimers undergo conformational changes followed by pY phosphorylation and activation of the receptor-associated tyrosine kinase JAK2. JAK2 mediates phosphorylation of the receptor at the cytoplasmic end at specific sites. STAT5 can bind to the receptors as preformed parallel and anti-parallel dimers regardless of its phosphorylation status and is activated by JAK2. The activated STAT5 proteins form parallel homo- or hetero (STAT5A/A; STAT5A/B; STAT5B/B) dimers via their SH2 domains and translocate to the nucleus to bind to the DNA. In *BCR-ABL1*-induced leukemias a translocation between chromosomes 9 and 22 occurs and results in a constitutive active cytoplasmic tyrosine fusion kinase. Depending on the break-point within the *BCR* gene three predominant variants of the *BCR-ABL1* kinase are known: p190^BCR-ABL1^, p210^BCR-ABL1^, p230^BCR-ABL1^. The associated leukemias are shown next to the different fusion kinases. *BCR-ABL1* activity rewires the JAK/STAT signaling in the leukemic cell. STAT5 is directly pY phosphorylated by BCR-ABL1 which makes JAK2 signaling dispensable. STAT5 tetramers and STAT oligomers (with activated STAT3 dimers) bind to the DNA and result in elevated target gene expression for the indicated cellular processes in a leukemic cell.

**Figure 2:**
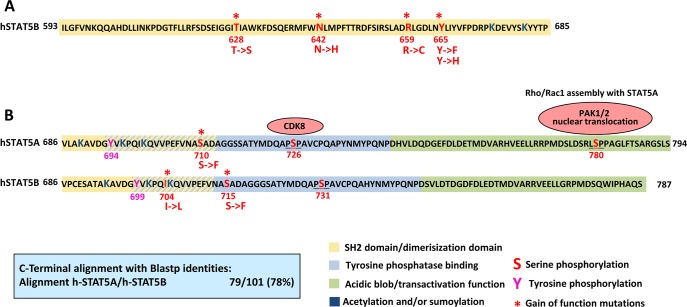
Gain of function mutations in STAT5 **(A)** The SH2/dimerization domain (yellow) of STAT5B ranges from 593 to 712 amino acids [105]. So far, somatic mutations in the STAT5B SH2 domain have been described in LGL, T-ALL, T-PLL and HSTL. Asterisks indicate the GOF mutation position. **(B)** The C-terminus of STAT5A and B is the most divergent part and shares 78% sequence identity between the two closely related proteins. Lysines (K- dark blue) nearby and in the tyrosine phosphatase binding domain (light blue) undergo acetylation or sumoylation, which positively or negatively regulates pYSTAT5, respectively [106]. Apart from tyrosines 694/699 (pink), serines sites (red) 726/780 in STAT5A are constitutively phosphorylated and crucial for leukemic transformation. As upstream kinases CDK8 and PAKs have been identified. GOF mutations have been described for S710/S715 in retro virally induced screening methods and I704 in T-ALL. The transactivation domain (green) is rich in aspartic (D) and glutamic acid (E) forming a highly negatively charged region, the acidic blob, which interacts with other factors of the transcriptional machinery.

## STAT5 biology

Only upon ligand binding to the cytokine receptor, the associated JAK kinase dimer becomes trans-activated and phosphorylates the cytoplasmic part of the receptor on distinct tyrosine residues [5]. Newest findings present a complete model of receptor-linked JAK2 activation after growth hormone (GH) binding [6]. Once the GH receptor dimer is activated, the transmembrane helices rearrange from a parallel to a left-handed cross-over state. This causes the removal of one JAK2 pseudokinase domain from the kinase domain of the respective JAK2 binding partner, trans-activation of the kinases and phosphorylation of the receptor. Another recent study enlightens the interaction between the JAK kinase, tyrosine kinase 2 (Tyk2) and the interferon-α receptor (IFNAR1) [7]. Binding to IFNAR1 resembles a SH2-like phosphopeptide interaction with Tyk2, with a glutamate replacing the usual phosphotyrosine residue when co-crystallized. STAT proteins bind via their N-terminus and SH2 domain to the phosphorylated cytokine receptors and crystal structure analysis revealed their pre-dimerization without the necessity of tyrosine phosphorylation as parallel/anti-parallel dimers [8]. Tyrosine phosphorylated STATs form efficient dimers via their SH2 domains and translocate to the nucleus to bind DNA. The two variants of STAT5 (STAT5A/B) are activated by more than 20 different cytokines, hormones and growth factors. Prominent cytokines include interleukin (IL)-2, 3, 4, 5, 7, 9, 15, 21, erythropoietin (EPO), thrombopoietin (TPO), prolactin (PRL), and granulocyte macrophage colony-stimulating factor (GM-CSF) and GH [5]. Activation is associated with tyrosine 694/699 phosphorylation in human STAT5A/B, which is a prerequisite for stable parallel dimer formation and initiation of transcription of STAT5-regulated genes [5]. Specific isoforms of STAT5A/B were associated with human cancer types, but the exact roles for each isoform in distinct cancer types are not studied yet [4]. Both proteins are widely expressed, but differences became also apparent in single knock-out mice. Loss of *Stat5a* results in impaired mammary gland development [9], whereas deletion of *Stat5b* causes stunted body growth and NK cell defects [10]. *Stat5a/b* double knock-out mice die perinatal on a C57BL/6 and Balb/c genetic background, but Sv129/C57BL/6 *Stat5a/b* double knock-out mice have a compensatory mechanism via high pYSTAT3 activity and a sub Mendelian fraction of severely sick mice can survive up to 5 weeks [11]. The reversible tyrosine phosphorylation of the STAT proteins is regulated by protein tyrosine phosphatases (PTP) of which 109 different family members are known [12]. SH2-domain-containing protein tyrosine phosphatase-2 (SHP-2), PTP1-B as well as serine protein phosphatase 2A (PP2A) have been reported to be associated with STAT5 regulation but it remains largely unknown which particular phosphatases act on STAT5A/B in specific cell types [13–15]. Interestingly, serine phosphatase PP2A-activating drugs were recently found to kill therapy-resistant chronic myeloid leukemia (CML) stem cells [16].

## STAT5, the central signaling node in *BCR-ABL1*^+^ leukemias

Persistent activation of STAT5 is found in many solid cancers as well as in the majority of hematologic malignancies [4]. Especially in myeloid malignancies, the role and oncogenic properties of STAT5 are well established [17–20]. The role of STAT5 proteins in solid cancers is less clear due to largely lacking conditional targeting approaches in appropriate genetic cancer models. With regard to most hematopoietic cancers, STAT5 is unmutated and its constitutive activation is a secondary event and triggered by oncogenic upstream tyrosine kinases such as BCR-ABL1 (Figure 1). The requirement of STAT5 in *BCR-ABL1^+^* leukemias for transformation and disease maintenance has been well described [21]. Further, the role of the different STAT5A/B isoforms has recently been addressed, where in a *BCR-ABL1*-inducible cellular system, knock-down of STAT5B sensitized leukemic cells to imatinib treatment [22] and the attenuation of STAT5A resulted in enhanced basal oxidative stress and DNA damage of normal CD34^+^ and CML cells as well as growth inhibition of CD34^+^ cells from patients with acquired imatinib resistance [23].

CML is a prototype of a myeloid neoplasm where a single driver mutant, BCR-ABL1, initiates activation of multiple pro-oncogenic signaling molecules, including STAT5 [24]. In the chronic phase of CML, BCR-ABL1 is essential for the survival and proliferation of leukemic cells. In line with this concept, BCR-ABL1-targeting drugs are able to produce stable complete responses in many patients with Philadelphia chromosome positive (Ph^+^) CML [25]. Indeed, for these patients, the development of BCR-ABL1 tyrosine kinase inhibitors (TKI) remains a huge success story. The first active compound with large clinical applications in CML was imatinib [26] followed by dasatinib, which binds BCR-ABL1 and other oncogenic kinases, such as SRC family kinases in both configurations of inactive or active kinase state [27]. Further TKI improvement came from the higher binding affinity and selectivity than imatinib through the follow-up substance nilotinib (AMN107) [28]. In addition, bosutinib (SKI-606) was developed, a second line inhibitor which has the broadest target spectrum inhibiting SRC, ABL and TEC, as well as serine kinases CAMK2G and STE20, but bosutinib does not target PDGFR and KIT [29, 30]. Finally, the third line inhibitor ponatinib has been developed and shown to exhibit substantial efficacy in patients with CML in whom the polyresistant *BCR-ABL1 T315I* mutation is expressed in leukemic cells [31].

## STAT5 enhances resistance to therapy

Overall, TKI have changed the fate of patients suffering from BCR-ABL1-driven CML [25] or Ph^+^ acute lymphoblastic leukemia. Despite this great success,TKI are not curative and in most cases medication needs to be taken life-long to prevent relapse [32]. On the other hand, CML might be cured with TKI in some cases as shown by the Stop imatinib (STIM) trial [33]. Within a cohort of 100 patients who were treated with imatinib for at least two years and had achieved a complete molecular remission (CMR), the probability to persist in CMR was 41%. Nevertheless, CML is a chronic hematopoietic disease derived from transformed long-term hematopoietic stem cells (LT-HSC) that can act as leukemic stem cells (LSC) and maintain the disease by signaling via STAT5 [34]. Majoritarian, TKI treatment results in the decimation of LSC below detection limits but is unable to completely eradicate the LSC [35]. This TKI resistance can be explained by the behavior of LSC like non-transformed stem cells rather than oncogene addicted CML cancer stem cells [36]. Further, CML stem cells are capable of autocrine TNF-α production which supports the expression of IL-3 and GM-CSF receptor and is associated with cell survival and enhanced proliferation [37]. Moreover, CD25/IL-2 receptor high expressing CML stem cells were shown to have a higher capacity to induce leukemia than CD25 negative CML stem cells [38]. Interestingly, a similar important role for STAT5-regulated CD25 expression as a leukemic stem cell marker was demonstrated to be a predictive biomarker in an acute myeloid leukemia (AML) for sensitivity of PIM kinase inhibitors [39]. These new findings further underline the role of STAT5 signaling in CML or AML stem cells.

15–25% of all patients develop resistance towards imatinib mostly mediated by point mutations of the *BCR-ABL1* oncogene [40]. In this regard it is of interest that high levels of STAT5 suffice to enhance imatinib resistance in CML cells [41]. High STAT5 expression levels were also shown to correlate with the occurrence of *BCR-ABL1* mutations [42]. Formal proof for STAT5 as proto-oncogene in hematopoietic diseases was obtained by retroviral transduction of normal murine bone marrow (BM) cells followed by transplant with gain of function (GOF) *Stat5* variants. Thus, hyperactivity of STAT5 as mimicked by expression of a constitutively activated STAT5A (cSTAT5A) variant suffices to induce a multilineage leukemia upon transplantation [43].

## Somatic mutations of STAT5 on the rise

Since 2013, somatic *STAT5B* mutations were discovered in large granula lymphocytic leukemia (LGL) [44], in T-promyelocytic leukemia (T-PLL) [45], in acute T-cell leukemia (T-ALL) [46,47] and hepatosplenic T-cell lymphoma (HSTL) [48], where more mutations can be expected through progress in the cancer genome project [200]. Over-expression of the mutated *STAT5B* versions resulted in increased transcriptional activity and was associated with poor disease outcome. Surprisingly and interestingly, most mutations occur in the SH2 domain of STAT5B, with the point mutation N642H being the most frequent. How these mutations might affect nuclear shuttling of STAT5 is unclear. A graphical scheme of GOF somatic *STAT5B* mutations is given in Figure 2A. In addition, in acute promyelocytic leukemia, *STAT5B* translocates and fuses with the retinoic acid receptor (RAR) alpha with resulting enhanced STAT3-mediated signaling [49]. cSTAT5A triggers leukemogenesis through the N-terminus, which regulates STAT5 tetramer formation and oligomerization among multiple STAT family members [43]. Furthermore, cSTAT5 in hematopoietic stem cells (HSC) was shown to be crucial for development of leukemia and knock-down of *STAT5A* was associated with inhibiting growth of CML CD34^+^ cell from patients with acquired resistances towards TKI [23,50]. Interestingly, in leukemic cells, tyrosine phosphorylated STAT5A (pYSTAT5) not only localizes in the nucleus but it is also found predominantly localized in the cytoplasm where it can form a signaling complex with GRB-associated binding protein 2 (Gab2) and phosphoinositide 3-kinase (PI3K) leading to protein kinase B (PKB) activation [51]. It is unclear and hard to dissect whether the cytoplasmic activity of STAT5 contributes to its oncogenic potential, but studying the effects of inhibitors of nuclear STAT5 transport could illuminate the role of cytoplasmic STAT5 in the context of leukemogenesis.

## Blocking STAT5 nuclear translocation

**Figure 3:**
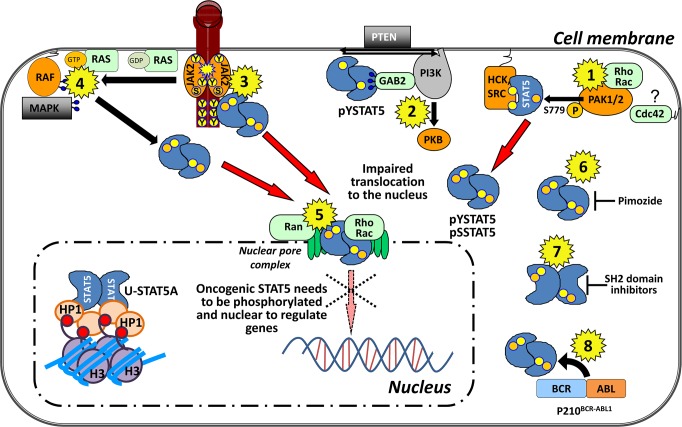
Targeting STAT5A nuclear translocation at a glance in leukemia **(1)** PAK1 and 2 phosphorylate STAT5A on S779 in the presence of Rho GTPases and might travel through the nuclear pore complex assembled to those. Here, targeting Rho GTPases and the down-stream effectors, PAKs, seem reasonable. Cdc42 is a close homologue of Rho, but it interacts with membranes via post-translational C-terminal geranylgeranyl lipid modification. It is unclear if Cdc42 is involved in shuttling processes of STAT5 (marked by a ?) and it is thus not found in the nucleus. SRC-family kinases (SFK) retain pYSTAT5A in the cytoplasm. Thus, targeting SFK could enhance STAT5 nuclear localization, which might not be therapeutically beneficial. **(2)** Targeting the complex formation of pYSTAT5A in the cytoplasm with Gab2 and PI3K which lead to PKB activation**. (3)** JAK2 inhibition targets the canonical signaling and nuclear translocation which was shown to be relevant in the CML stem cell. **(4)** Targeting oncogenic Ras which promotes nuclear localization of STAT5 in HSC. **(5)** Ran GTPases are thought to be essential mediators of the STAT-transporter complex involved in nuclear translocation. **(6)** Pimozide inhibits pYSTAT5. **(7)** SH2 domain inhibitors block the effective dimer formation of STAT molecules that bind to DNA. **(8)** BCR-ABL1 phosphorylates STAT5 directly thus TKI target tyrosine phosphorylation and nuclear translocation of STAT5 indirectly.

Several lines of evidence argue for a critical role of STAT5A/B in leukemia. Based on the essential role of these molecules in leukemia evolution and progression, several targeting approaches have been proposed [52, 53]. We could show that serine phosphorylation of STAT5A is a prerequisite for nuclear translocation [54]. Here, we propose that the inhibition of this transport by direct or indirect interaction with the transcription factor or upstream regulators of nuclear import (summarized in Figure 3) could be a feasible targeting approach. Unbiased cell-based screens with various small-molecule compound libraries are one way to identify new targets of already approved drugs. By employing STAT5 transcriptional activity and luciferase reporter assays, pimozide was identified as a potential STAT5 inhibitor in *BCR-ABL1*^+^ leukemic cells and in an AML mouse model [55, 56]. Pimozide was found to inhibit tyrosine phosphorylation of STAT5, but did not target BCR-ABL1. The mechanistic details of STAT5 inhibition by pimozide are unclear but most likely indirect. A direct interaction between STAT5 and pimozide was not demonstrated and the use of high molecular concentrations suggests that the effects of pimozide on STAT5 might be secondary and not potent enough to be considered for clinical application [55]. Still it was the first experimental tool to target STAT5 in clinically relevant cell systems. A current study identifies the synthetic chalone α-Br-TMC as an inhibitor of JAK2/STAT5 signaling [57]. Although, cytotoxic effects are only observed at high concentrations (100 μM) in cells expressing cSTAT5, tyrosine phosphorylation of STAT5 and JAK2 was strongly reduced at 10 μM. STAT5 dependant target gene transcription is altered but is remains to be determined whether STAT5 inhibition is direct or a secondary effect.

## SH2 domain inhibitors

The transcriptional activity of STAT5 requires phosphorylation on tyrosine and parallel dimer formation via SH2 domains. Inhibitors targeting the SH2 domain of STAT5B have been identified but very high concentrations of these compounds were required to induce apoptosis in lymphoma cells [58]. In a report by the Gunning group salicylic acid-based small molecules targeting STAT5 SH2 domain also required high IC50 values to counteract growth of *BCR-ABL1^+^* cells (20 μM) [59]. The same group improved STAT5 SH2 domain inhibitors further that now work *in vitro* in a nanomolar range (Cumaraswamy et al. 2014 accepted in ACS Medicinal Chemistry Letters). Of interest is also a STAT3 SH2 domain-targeting compound that showed low micromolar effects (1 μM) in combination with imatinib on survival of CD34^+^ cells obtained from CML patients with BCR-ABL1-independent TKI resistance [60]. This is of clinical importance as STAT3 was shown to mediate resistance in these leukemias.

## Up-stream regulator JAK2

The receptor-associated tyrosine kinase JAK2 is the main activator of STAT5 in hematopoietic cells and is thus considered an obvious target in JAK-STAT-driven malignancies. Further, the identification of activating mutations in JAK kinases in Ph^−^ myeloproliferative neoplasms, with JAK2 (V617F) being the most frequent [61–63], has changed therapeutic strategies dramatically. Moreover, many JAK mutations were shown to be associated with acute leukemias [64,65]. Indeed, JAK-targeting TKI have reached the clinics recently, several of which also target JAK2 with low micromolar concentration to exert pro-apoptotic effects in CML cells *in vitro* (reviewed in [66]). However, *BCR-ABL1*-induced myeloid transformation and disease maintenance is independent of JAK2 in mouse models and STAT5 is highly activated despite deletion of JAK2 in these leukemia models [67]. Off-target effects of JAK2 TKI inhibitors explain this apparent contradiction. Most JAK2-targeting TKI are capable of inducing apoptosis not only in JAK2 wild-type cells but also in JAK2-deficient cells by inhibiting BCR-ABL1 kinase itself. Transformation by *BCR-ABL1* rewires signaling and BCR-ABL1 itself phosphorylates and activates STAT5. Another layer of complexity was introduced by the fact that knock-down of *BCR-ABL1* in CD34^+^ human CML stems cells fails to efficiently eradicate these leukemic cells [68]. Reduced colony formation of CD34^+^ CML cells in the presence of dasatinib was complemented upon administration of cytokines that provide survival signals via JAK2 [69]. On the other hand, the absence of JAK2 dramatically accelerated disease progression in a *BCR-ABL1^+^* mouse model [70]. Competitive transplantation studies showed that loss of JAK2 enabled leukemia initiating cells to outcompete the normal HSC. This again relativizes the possible benefit of JAK2 inhibition in *BCR-ABL1^+^* leukemia, as the kinase is a critical factor in normal hematopoiesis. Thus, JAK2 inhibition might affect HSC and LSC in comparable manner and even abolish the therapeutic efficacy [71]. Nevertheless, early clinical trials aim to assess the possibility to combine the JAK2 inhibitor ruxolitinib in CML patients receiving already treatment with TKI [201, 202]. A recent study demonstrated reduction and apoptosis of CD34^+^ CML cells upon combined treatment with nilotinib and ruxolitinib, rather than single application [72]. In the mouse model the combination of TKI showed toxicity toward healthy HSC, but to a lesser extent than toward CD34^+^ CML cells, which suggested that combinatorial TKI therapy is advantageous over TKI monotherapy.

## Controversial: targeting SFK

Other kinases that have been reported to activate STAT5 in *BCR-ABL1*^+^ disease belong to the SRC-family kinases (SFK). The SFK members HCK and LYN were found to be highly activated in p210^BCR-ABL1^ cells [73]. SRC interacts with STAT5 and can phosphorylate STAT5 on tyrosine 694 upon EPO receptor stimulation [74]. Similarly, HCK is capable of phosphorylating STAT5B in *BCR-ABL1*-transformed cells [75]. The effects of the BCR-ABL1 TKI dasatinib, which also inhibits SRC kinase activity and the dual SRC/ABL kinase inhibitor bosutinib on CML stem cells appeared mild and no strong pro-apoptotic response could be achieved [76,77]. Recently, the SRC kinases HCK and SRC, but not LYN, were shown to retain STAT5A in the cytoplasm of *BCR-ABL1^+^* cells [78]. The significance of this observation is indicative of a potential negative control of SFK to retain pYSTAT5 in the cytoplasm blocking or causing diminished nuclear pYSTAT5-induced transcription. Thus, SFKs might balance the high levels of STAT5 which was shown to associate pretty much in a 1:1 stoichiometry in K562 BCR-ABL1^+^ cells to relieve oncogenic stress induced by STAT5 [67,79]. Thus, inhibition of this control mechanism by targeting SFKs might explain partly why SFK inhibitors failed for clinical applications since their inhibition could accelerate pYSTAT5 nuclear translocation.

## STAT5 serine phosphorylation

STAT5 harbours serine phosphorylation sites within the C-terminal TAD, with only partially understood biological functions (reviewed in [80]). Both, STAT5A and STAT5B harbour a serine residue at position 725 or 730 within a conserved PSP motif that becomes phosphorylated upon stimulation with GH, PRL and IL-2 (see graphical scheme Figure 2B). Mutation of the conserved serine site was shown to negatively influence transcriptional activity of STAT1 and STAT3. Interestingly, human STAT5A has a unique serine site at position 780 within an LSSP motif. The role of those serine sites in *BCR-ABL1^+^* leukemia has recently been clarified. STAT5A is highly serine-phosphorylated in human CML cells. *Friedbichler et al*. showed that loss of serine 725 and 779 phosphorylation impaired leukemogenesis induced by cSTAT5A [81]. The fact that regular hematopoiesis was largely unaffected placed STAT5 serine phosphorylation into the lime light of potential target sites.

In fibroblasts, the serine/threonine kinase inhibitor flavopiridol reduced STAT5 serine 725 phosphorylation triggered by the growth-factor GM-CSF and knock-down experiments identified CDK8 as a responsible up-stream kinase phosphorylating STAT1 on serine 727 [82]. In leukemias this STAT1 serine site is also critical as natural killer (NK) cells lacking phosphorylation on STAT1 serine 727 displayed enhanced cytotoxicity and were capable to eradicate leukemic cells significantly better when compared to wild-type STAT1-expressing cells [83]. Treatment with CDK8 inhibitors could thus improve tumor surveillance by enhancing NK cell-mediated cytotoxicity. Flavopiridol was the first serine/threonine kinase inhibitor in clinical trials and is currently tested in clinical chronic lymphocytic leukemia (CLL), AML, and CML trials. A Phase I clinical trial with combination therapy consisting of imatinib and flavopiridol in CML and *BCR-ABL1^+^* ALL patients has already been conducted with considerable success [84].

## Targeting p21-activated kinases

In *BCR-ABL1*^+^ leukemias, STAT5A mutants lacking serine phosphorylation on S725 and S779 strongly impaired the viability of the leukemic cells and did not support transformation by Abelson oncogenes [54]. The mutation of a single serine (STAT5^S779A^) sufficed to prolonged disease onset in BCR-ABL1-driven leukemic mouse model. We identified p21-activated kinases (PAK) as major up-stream kinases and thus triggers of pSTAT5^S779^. Biochemical studies confirmed the direct interaction and phosphorylation of STAT5^S779^ by PAK which required the concomitant presence of the Rho GTPase family [54]. Further evidence for a key role of PAK in hematopoietic malignancies stems from a report on c-kit-induced myeloid neoplasms where PAK acts in conjunction with Rac kinase to support leukemogenesis [85]. Interestingly, Rac-dependant signaling has also been implicated in the signaling cascades downstream of p210^BCR-ABL1^ in CML stem and progenitor cells [86–89]. Rac1 and its up-stream activator MgcRacGAP, a nuclear localizing signal-containing nuclear chaperone, are essential for the nuclear translocation of STAT transcription factors in cytokine-induced and oncogenic (Flt3-ITD) signaling [90,91]. Our recent work bridges the gap and allows us to understand the mechanistic details underlying these observations. In particular, STAT5^S779^ phosphorylation has been identified as a prerequisite for STAT5A to translocate into the nucleus and nuclear accumulation of STAT5A is prevented by PAK I group kinase inhibitors in *BCR-ABL1^+^* leukemias [54]. The importance of STAT5^S779^ for nuclear translocation has long not been recognized perhaps because it has been masked by endogenous wild-type STAT5 [92–94]. We introduced a YFP-tagged STAT5^S779A^ mutant into BCR-ABL1-expressing HEK 293T cells and observed no accumulation in the nucleus but an even distribution throughout the cell [54]. Upon co-transfection with untagged STAT5 wild-type, nuclear accumulation of YFP-tagged STAT5 was observed. Thus, with a STAT5 molecule with intact S779 within the dimer, transport to the nucleus is possible. This might also explain why dominant-negative STAT5 lacking the TAD with the serine site 779 is still able to go nuclear [95]. Further, the massive over-expression might also promote STAT5 into the nucleus. Human STAT5B also harbours a serine site at position 779 in the TAD but it shows essential differences to STAT5A as the motif is not flanked by prolines (DSQ) [102]. The role of this phosphorylation site has not been addressed so far. The mechanistic details of STAT5B translocation to the nucleus remain unknown, but it is generally accepted that STAT5B can be transported to the nucleus in a heterodimer with STAT5A and upon GH stimulation STAT5B homodimers efficiently go nuclear [96].

## GTPases and STAT cellular shuttling

STAT proteins were also shown to be able to undergo nucleocytoplasmic transport through the cell without being tyrosine phosphorylated (U-STAT), whereas the re-translocation of pYSTAT from the nucleus back into the cytoplasm requires its dephosphorylation. In order to translocate into the nucleus, molecules need to pass the nuclear pore complex (NPC) which consists of about 30 different proteins, the nucleoporins (Nups) (reviewed in [97] and [98]). Large proteins such as the STAT proteins (95-100 kDA) cannot freely pass the NPC. Two modes of transfer through the NPC have been postulated: (i) carrier-independent transport mediated by direct interaction with the Nups or (ii) carrier-dependent transport, where the cargo protein is associated with the transport factors importins for nuclear import and chromosome region maintenance 1 (CRM1) protein for export from the nucleus. The Ras-related nuclear (Ran) proteins are present in the cell in two nucleotide bound forms: GDP- and GTP-bound. The asymmetrical distribution of Ran-GTP, which is highly concentrated in the nucleus and Ran-GDP, with high levels in the cytoplasm, drives nuclear shuttling. Non-tyrosine phosphorylated STAT proteins were shown to travel by carrier-free transportation mediated by direct interactions with the Nups as well as through carrier-dependant transport out of the nucleus mediated by interaction with CRM1 [99]. For nuclear export of STAT proteins, a ternary complex of CRM1-RanGTP with a Leucine-rich nuclear export signal needs to be formed in the nucleus and this complex is then disassembled by GTP hydrolysis by Ran GTPase activating protein in the cytoplasm. These shuttling processes are dependent on small GTPase activity which seems to be in line with the finding that phosphorylation of STAT5A on serine 779 by PAKs requires the presence of members of the Rho GTPase family which might travel in complex with the STAT5 proteins through the nuclear pore. This implies that translocation of STAT5A is regulated by small GTPases, that can be therapeutically targeted with small molecule compounds. However, we want to mention that small GTPases such as oncogenic Ras are difficult to be targeted due to the high affinity of GTP to small GTPases [100]. Interestingly, oncogenic Ras was recently linked to promote enhanced nuclear accumulation of STAT5 in HSC, partly through diminished *Socs2* expression promoting renewal and engraftment under competitive transplant situation and accelerated growth of HSC and the myeloid progenitor pool in particular [101]. Inhibitors targeting Rho GTPase family members and their downstream effectors have been developed [102,103] and only a few were adopted for clinical use and therefore data on toxicity in mammals are limited (reviewed in [104]).

## CONCLUSIONS

Treatment of TKI-resistant CML is an emerging challenge in experimental and applied hematology. In fact, CML LSC exhibit multiple forms of resistance and may escape treatment with novel TKI. This has accelerated the search for alternative therapies and drug targets in CML. As described in this article, STAT5 fulfils all criteria of a major drug target in *BCR-ABL1*^+^ leukemia. However, there are other potential indications for STAT5 inhibitors. Notably, STAT5 is important for efficient generation of many blood lineages and it is essential for proper immune function. Moreover, it is an important regulator in function and differentiation of epithelial cell types. It can be a driver or preventer of solid cancers and STAT5 protein ablation might not turn out favourable, but it was surprisingly well tolerated in genetic mouse models of *Stat5a/b* ablation. The path to therapeutic success might involve the targeting of modulators such as serine kinases or small GTPases and interacting proteins of STAT5. The specific inhibition of components of the aberrant signaling to which STAT5 is a central mediator can result in differentiation, growth arrest or apoptosis of leukemic cells. Thus, modulation of the irregular signaling in the cancer cell towards the normal levels is a promising avenue to target STAT5-dependent leukemia such as CML.

Formerly, transcription factors like the STAT family members were considered to be undruggable as they lack a catalytic activity. Today, there are many possibilities to target STAT proteins indirectly via interfering with its up-stream regulators thereby negatively influencing the nuclear translocation of STAT proteins and inhibiting their transcriptional activity. The direct inhibition via SH2 domains has been a long discussed issue considering that formerly developed SH2 domain inhibitors lacked potency and specificity. However, continuous improvements were achieved in this field and the development of the first inhibitor displaying low micromolar antileukemic potency might soon have consequences for the clinics.
